# Bibliographical

**Published:** 1856-04

**Authors:** 


### BIBLIOGRAPHICAL.

We have received from Messrs. Samuel S. & W. Wood, 261 Pearl street, New York, a number of valuable medical works, and for the convenience of those who would like to have them sent *by mail*, direct from the publishers, we attach the prices for which they can be obtained, *free of postage.*

First upon the list, we find "Clinical Lectures on the diseases of Women and Children," by Gunning S. Bedford, A. M. M. D., Professor of Obstetrics, the Diseases of Women and Children and Clinical Midwifery, in the University of New York. Though this work has been subjected to a severe criticism, the fact that the first edition was exhausted in a period of three months, is conclusive evidence of the high estimate put upon it by the profession, and we cordially add our testimony to its value as an eminently practical work, based upon the most approved physiological principles; and as a specimen of the style of the work, in an article on the Diseases of the Nervous System in Infancy, after giving a physiological explanation of the first respiratory movements, he remarks, "From this you derive a most important therapeutical principle, viz: that the remedy for asphyxia in the new-born infant is the prompt stimulation of the peripheral extremities of the respiratory nerves, so through the effects of this stimulation on the spinal cord, a motor impulse may be imparted to the respiratory muscles. This will ensure their contraction, and on this depends the act of respiration. You see, therefore, that the first act of independent life in the infant, respiration, is derived from the spinal cord, and also the first disease of the new-born infant is the result of inaction of this same nervous centre.

"But let us proceed a step further, and we shall have abundant evidence that in the investigation of the nervous affections of infancy, we should be in the constant commission of error, if we lose sight of that important nervous centre—the spinal system.

"One of the great facts of modern physiology—a fact which has removed the obscurity which formerly existed, and which has led to sound therapeutical applications, is this—that in all convulsive diseases the spinal cord is more or less involved, or in other words, that spasmodic affections cannot exist, other than as the effect of derangement, either organic or functional, of the spinal system.

"What a precious fact, and what a contrast does it institute between the physiology of the present and the past! If, however, we have a better physiology now than formerly, or if the laws of this beautiful science are better understood, it follows as a necessary consequence, that we must have a sounder and more rational therapeutics; for to the medical man the value of physiological principles is in direct ratio, to the aid they afford him in the treatment of disease."

We could wish that we had more space for other extracts, but hope that all will own the work; it contains 567 pages, and can be had of the publishers at $2.75.

We next have "Organic Diseases and Functional Disorders of the Stomach," by George Budd, M. D., F. R. S., Professor of Medicine in King's College, London, &c., containing 283 pages of valuable matter, on self-digestion of the stomach, or changes that take place in the coats of the stomach after death, from the action of the gastric juice—a change manifested to some extent, in a large proportion of post mortem examinations, without a knowledge of which, it is apparent, that we should not be able to discriminate between the results of disease, and changes which may have taken place after dissolution, and as the author remarks, a study of this is not only a

4

necessary preliminary to the study of the diseases of the mucous membrane, but it also throws light on the circumstances which promote the secretion of the gastric juice, and on various functional disorders to which the stomach is liable.

Nearly fifty pages are devoted to the important and little understood subjects of congestion and inflammation of the stomach; some thirty or more to ulceration of the stomach and duodenum; fifteen or twenty to cancer of the stomach, making up about half the work; the remainder being devoted to sympathetic disorders of the stomach from irritations elsewhere, deficient secretions of gastric juice, fermentation in the contents of the stomach, with development of sarcenæ, indigestion arising from impurity of the blood, urticaria, Pyrosis, gastralgia, indigestion of drunkards, vomiting, excessive acidity, and flatulence, with remarks upon some of the remedies for stomach disorders, including ipecacuanha, bismuth, the vegetable astringents, hydrocyanic acid, alkalies, mineral acids, vegetable bitters, preparations of steel, purgatives, and general rules of living.

This work can be had of the same publishers, at $1.50 *by mail free of postage.*

Also, the "Practitioner's Pharmacopœpia and Universal Formulary," containing 2000 classified prescriptions, selected from the practice of the most eminent British and Foreign medical authorities, with an abstract of the three British Pharmacopœias and much other useful information for the practitioner and the student, by John Foote, M. R. C. S., London, with corrections and additions, by an American physician, price, $1.50, as above.

The well known and popular Medical Lexicon of (now in its third edition,) D. Meredith Reese, M. D. L. L. D., being a complete vocabulary of definitions, including all the technical terms employed by writers and teachers of medical science, at the present day. Price, 50 cents.

The "Anatomical Remembrancer, or Complete Pocket Anatomist," containing a concise description of the structure of the human body, by C. I. Isaacs, M. D., Demonstrator of Anatomy, in the University of New York. Price, 50 cents.

And a little book of 69 pages, which professes to teach "How to nurse sick children," and intended to be beneficial to all who have charge of the young, which is doubtless well adapted to the end proposed. Price, 37½ cents. With a bound catalogue of 150 pages, containing a list of medical books for sale by S. S. & W. Wood, furnished *gratuitously*, *post paid*, to all who apply.

